# Prevalence and risk factors associated with postnatal depression in a South African primary care facility

**DOI:** 10.4102/phcfm.v12i1.2538

**Published:** 2020-11-27

**Authors:** Nyundu S.J. Phukuta, Olufemi B. Omole

**Affiliations:** 1 Division of Family Medicine, Department of Family Medicine, Faculty of Sciences, University of The Witwatersrand, Johannesburg, South Africa

**Keywords:** postanal, depression, primary care, prevalence, risk factors

## Abstract

**Background:**

The prevalence and factors that influence postnatal depression (PND) vary according to context.

**Aim:**

To determine the prevalence and factors associated with PND in the postnatal clinic of a large community health centre.

**Setting:**

This study was conducted at Levai Mbatha Community healthcare centre, in Evaton, South of Gauteng.

**Method:**

In a cross-sectional study, the Edinburg Postnatal Depression Scale (EPDS) was administered on 227 consecutive mothers during postnatal clinic visits. In addition, sociodemographic and clinical information were collected. Analysis included descriptive statistics, chi-square test and logistic regression. A score of greater than 13 on the EPDS screened positive for PND.

**Results:**

Participants’ mean age was 27 years, and most completed less than grade 12 education (52.4%), were single (55.5%), were employed or had a working partner (60%) and had no previous PND (97%). The proportion of participants screening positive was 38.8%. In the adjusted logistic regression, completing only primary school education (odds ratio [OR]: 9.11; 95% confidence interval [CI]: 1.03–80.22; *p* = 0.047), using contraceptive prior to index pregnancy (OR: 2.05; 95% CI: 1.12–3.72; *p* = 0.019) and reporting a thought of self-harm or infanticide (OR: 7.08; 95% CI: 5.79–22.21; *p* = 0.000) significantly increased the risk of PND. In contrast, having a relationship with the father of the index child (OR: 0.42; 95% CI: 0.18-0.94; *p* = 0.037) mitigated this risk.

**Conclusion:**

The proportion of women screening positive for PND was high in the study setting and was concomitant with significant risk of suicide or infanticide. This highlights the need to screen and consider PND as a vital sign during postnatal visits, especially in the face of low educational attainment, failed contraception and poor or no relationship with the father of the index child.

## Introduction

Depression is an important cause of disability and constitutes a large proportion of the global burden of diseases.^1^ Women of reproductive age are amongst the most affected, especially during the perinatal period.^2^ If undiagnosed and untreated, depression amongst pregnant women is associated with poor quality of life for the mother, child and the family, and increases the risks of maternal suicide, infanticide and chronic depressive disorder.^3^

Worldwide, approximately one in five women will experience an episode of depression during pregnancy and/or in the postnatal period; the latter is usually defined as the first 6 weeks after delivery. However, depressive symptoms can occur beyond the 6-week period, and up to 20% of mothers develop depression by 16 weeks post-delivery.^4^ The Centre for Disease Control and Prevention reported in 2009 that 10% – 15% of women in the United States complained of signs and symptoms of postnatal depression (PND). This number is considerably higher in low- and middle-income communities, with most women in this population remaining undiagnosed.^5^ The prevalence of PND shows a wide range in developing countries – from 5% in Nepal to between 5% and 59% in India.^6^ The pooled prevalence of postnatal depression in low- and middle-income countries is estimated to be about 19.8%, which is higher than in developed countries.^7^ In Africa, PND is the most prevalent psychological disorder in the perinatal period, with a pooled prevalence of 18.3%.^7^ In South Africa, whilst the prevalence of PND may be as high as 34.7% in a peri-urban area,^3^ a recent study reported a higher prevalence of 50.3% in a rural setting.^8^ Although PND affects low-income countries disproportionately, most of the available data on PND come from studies in high-income countries.^7,9,10^ Even in South Africa, most data on PND emanate from studies conducted in the Western cape province, highlighting the need for data from other parts of the country. This is more so considering that differences in contexts may limit the generalisation of study findings across settings. This study therefore aimed to determine the prevalence and factors associated with PND in a peri-urban primary care facility, situated in a socio-economically deprived area, south of Johannesburg.

## Methods

### Study design and setting

This research utilised a cross-sectional design and was conducted at Levai Mbatha Community Health Centre (CHC) in Evaton Township, south of Johannesburg. This CHC renders 24-h emergency and maternity service, and other primary healthcare (PHC) services such as immunisation, primary mental healthcare services, primary oral health services, chronic diseases care, medical male circumcision program, allied and rehabilitation healthcare services and youth-friendly services. Its catchment population is 80 963 residents, of whom more than 80% are uninsured and depend on free public PHC.^11^ Postnatal and child healthcare are amongst the services that the PHC section of the clinic offer. Mothers who attend for their postnatal visits have mostly been delivered at the in-house Midwife Obstetrics Unit (MOU) or at the regional specialist hospital situated about 11 kilometres away.

### Study population

According to the District Health Information System, an average of 405 women consulted at Levai Mbatha CHC for postnatal consultation every 3 months at the time of the study.

### Sample size and sampling

Assuming a confidence interval of 95%, power of 80% and a margin error of 5% with a response distribution of 50%, we determined the minimum sample size required to be 198 postnatal mothers. We increased this by 15% to adjust for potential incomplete or missing data, determining a final sample size of 227.

Consecutive mothers, 18 years and older, up to 16 weeks postpartum, who came to the postnatal clinic or the baby clinic were approached individually after their vital signs were taken. The objectives and processes of the study were explained to these mothers as well as the possibility of them being referred to another healthcare practitioner in the mental healthcare section of the clinic for further assessment and management, if deemed necessary. The mothers were then invited to participate, and those who agreed were taken to a separate room where they were presented with the participant information sheet. Those who agreed to participate then signed the informed consent. Mothers who presented in the emergency department, mothers of stillborn babies or mothers of hospitalised babies were excluded. Over 3 months, we consecutively approached 236 postnatal mothers, of whom five refused to participate. Of the 231 postnatal mothers who agreed to participate, four were excluded due to participants not meeting inclusion criteria, leaving 227 for analysis. This brought the response rate to 98%.

### Tool and data collection

The data collection tool consisted of two sections:

The first was the Edinburg Postnatal Depression Scale (EPDS). The EPDS is the most used and internationally recognised tool for screening for PND and has been validated in South Africa.^12,13^ It consist of 10 questions that assess the mother’s mood, interest in pleasurable activities, self-blaming, worrying for no reason, panicking for no reason, being unable to control things, sleeping difficulty, feeling sad, crying for no reason and self-harm thoughts. The score for positive screening for PND is set at > 13.^14^

The second section was a structured questionnaire that collected information on socio-demographic and medical information, infant characteristics, delivery details of index baby and mother’s relationship status. In addition, this questionnaire probed for the presence of risk factors for PND enumerated in the literature.^3,15,16,17^

After obtaining informed written consent, the researcher or a trained assistant administered the data collection tools on participants. Each copy of the data collection tool had a unique anonymous code representing the participant’s study number. Participants who were screened positive for PND or those who scored more than zero for Question 10 of the EPDS, which screens for suicidal ideation, were immediately referred to the mental health section of the clinic for further assessment and management. The researcher collected completed EPDS and questionnaires at the end of every business day for data capture.

### Analysis

Data were captured onto MS Excel sheet and imported into STATA statistical analysis software, version 10 for analysis. Descriptive statistics were used to summarise participants’ socio-demographic and clinical characteristics, and to determine the proportion of mothers who screened positive for PND. Participants were then divided into two groups: those positive for PND (score > 13) and those negative (score 13 or less). Group differences in terms of socio-demographic and clinical characteristics were explored using the chi-square test. Where group differences and associations were statistically significant (*p* < 0.05), further regression analyses were carried out to determine the strengths of the associations.

### Ethical consideration

Before conducting the study, ethical clearance was obtained from the University of Witwatersrand Human Research Ethics Committee (HREC Medical), M160930. Permission was also obtained from the Sedibeng District Research Committee and Levai Mbatha CHC management.

## Results

A total of 227 participants were included in the study. The mean age was 27 years, and most were < 30 years old (70.5), single (55.5%), completed primary education or less (52.4%) and were either employed or had an employed partner (59.9%). Most participants reported a monogamous and supportive relationship with the father of the index baby and/or in-laws (Table 1).

**TABLE 1:** Socio-demographic and relationship characteristics.

Age group of mothers	Number	%
18–25 years	104	45.8
26–30 years	56	24.7
31–35 years	45	19.8
> 35 years	22	9.7
**Marital status**		
Single	126	55.5
Cohabiting	58	25.6
Married	42	18.5
**Level of education**		
Did not complete primary school	9	4.0
Completed primary school	110	48.5
Completed secondary school	91	40.1
Attended tertiary institution	17	7.5
**Baby had at least one parent employed**		
Yes	136	60.0
No	91	40.1
**Mother was in a relationship with the father of the child**		
Yes	194	85.5
No	33	14.5
**Mother was living with the father of the child**		
Yes	115	50.7
No	112	49.4
**Mother’s partner was financially supportive**		
Yes	193	85.0
No	34	15.0
**Mother was in a polygamous relationship**		
Yes	34	15.0
No	193	85.0
**Mother was receiving support from her family or in-laws**		
Yes	168	74.0
No	59	26.0

Most participants had no family history of mental health disorder (90.3%) and did not have any mental health problems (98.2%), previous history of PND (96.5%) or previous history of medical problems (67.8%) (Table 2).

**TABLE 2:** Perceptions of pregnancy, infant characteristics and delivery details.

Variables	Number	%
**Mother had planned the pregnancy**		
Yes	96	42.3
No	131	57.7
**Mother had a negative perception of the pregnancy**		
Yes	84	37.0
No	143	63.0
**Parity**		
Primipara	35	15.4
Multipara	192	84.6
**Desired sex**		
Yes	151	66.5
No	76	33.5
**Mother’s perception that baby cries often**		
Yes	49	21.6
No	178	78.4
**Mothers with a sick child**		
Yes	18	7.9
No	209	92.1
**History of miscarriage**		
Yes	39	17.2
No	188	82.8
**Mode of delivery**		
Normal vertex delivery	159	70.0
Caesarean section	68	30.0
**Mother had complications during labour**		
Yes	51	22.5
No	176	77.5
**Baby’s outcome after delivery**		
Good	211	93.0
Bad	16	7.1

Most participants did not plan the index pregnancy (57.7%), delivered by normal vaginal delivery (70.0%), had no complications during the index pregnancy (77.5%) and had good outcome of delivery (93%). Participants’ perceptions of the index pregnancy and baby were mostly favourable.

Approximately 38.8% of participants screened positive for PND. In addition, 32.5% of participants scored between 1 and 3 on Question 10 of the EDPS – ‘The thought of harming myself or my baby has occurred to me’.

On test of association, completing only primary education (*p* = 0.02), breast-feeding (*p* = 0.03), failure of contraceptive use (*p* = 0.03), reporting a negative perception of the pregnancy (*p* = 0.04), relationship with the father of the baby (*p* = 0.02) and a score on Question 10 of the EDPS (*p* = 0.00) were all significantly associated with PND.

In the adjusted multivariable regression analysis (Table 3), completing only primary education, using a contraceptive before the index pregnancy and scoring 1 to 3 on Question 10 of the EPDS independently increased the risk of PND. However, being in a relationship with the father of the index child independently mitigated the risk of PND.

**TABLE 3:** Results of adjusted multivariable analysis.

Variables	Odds ratio		95% CI	*p*
**Level of education**				
Did not complete primary school	1 (ref)	-		-
Completed primary school	9.11	1.034 – 80.22		0.047
Completed secondary school	3.82	0.43 – 34.12		0.230
Attended a tertiary institution	5.58	0.51 – 60.92		0.159
**Mother breastfeeding**				
No	1 (ref)		-	-
Yes	0.45		0.18 – 1.10	0.081
**Mother used contraceptive methods that failed prior to falling pregnant**				
No	1 (ref)		-	-
Yes	2.05		1.12 – 3.73	0.019
**Mother had negative perception of the pregnancy**				
No	1 (ref)		-	-
Yes	1.45		0.78 – 2.71	0.242
**Mother had anxiety during pregnancy**				
No	1 (ref)		-	-
Yes	1.27		0.67 – 2.40	0.467
**Mother was in a relationship with the father of the child**				
No	1 (ref)		-	-
Yes	0.42		0.19 – 0.95	0.037
**Mother’s core on Question 10 of the EPDS**				
0	1 (ref)		-	-
1–3	7.08		5.79 – 22.21	0.000

CI, confidence interval; EPDS, Edinburg Postnatal Depression Scale.

## Discussion

This study found that a high proportion of mothers (38.8%) screened positive for PND in this primary care setting. In addition, low educational attainment, use of contraceptive prior to the index pregnancy and a thought of harm to self or baby were significantly associated with increased risk of screening positive for PND. However, reporting an ongoing relationship with the father of the index baby mitigated this risk. The proportion of postnatal mothers screened positive for PND in this study is higher than reported elsewhere.^18,19,20^ This high proportion of positive screen like the prevalence of PND may be attributable to the pervasive poor socioeconomic conditions and high social stressor levels, typical of the current study setting. These factors have been associated with PND in several studies conducted in developing countries, including South Africa.^1,8,9,19^ Considering that this study found a high proportion of mothers screening positive for PND using a very sensitive tool such as EPDS – almost triple that reported in studies elsewhere^18,19^ – there is an urgent need to implement health policies that promote routine screening for PND.^21^ Because the symptoms of PND might have started before delivery, screening should also be done during antenatal care visits.^22,23^ As this was a study conducted in only one healthcare facility and employed a screening tool, a study with a bigger sample size using a diagnostic tool is needed to provide a clearer picture of the prevalence of PND and its related factors in South African primary care.

In this study, those mothers who only completed primary school were independently associated with positive screening for PND, consistent with findings from other studies conducted in developing countries.^9,24^ Low educational attainment is associated with socioeconomic deprivation and unemployment, and both have been reported to increase stress levels amongst South African mothers in the perinatal period, exposing them to a risk of depression.^21^ Addressing these links requires a multifaceted approach beyond screening and therapy in the health facility. Social and educational interventions are needed to improve women’s educational attainments, skills acquisition and emancipation.

The association between use of contraception prior to conception in the index pregnancy and PND can be explained as follows: firstly, this could have been due to contraceptive failure resulting in unplanned pregnancy – corroborated by the finding that more than half of participants in this study reported not planning the index pregnancy. Unplanned pregnancy on its own has been reported as a source of psychosocial stress and associated with an increased risk of PND in both developing and developed countries.^8,21^ There is therefore a need for healthcare workers to screen for PND amongst women who report that their pregnancies were due to failed contraception. Secondly, certain types of contraceptives such as hormonal ones increase the risk of developing PND.^22^ However, we did not inquire about the type and duration of contraceptive use, and we cannot ascribe the study findings to this relationship. Future studies need to fill these gaps.

That an ongoing relationship with the father of the index child is associated with lower risk of being screened positive for PND is consistent with previous reports elsewhere and in South Africa.^23,25^ A large cohort study in Soweto using the Pitt Depression Questionnaires (PDQ) found that difficulty in relationship with the partner and/or the father of the index child was a significant risk factor and almost doubled the risk of PND.^26^ Another study in rural South Africa also found that reporting a ‘None’ satisfaction regarding the romantic relationship between a woman and the father of her index child was associated with PND.^21^ Strenuous relationships with the index child’s father may negate financial and emotional support from the father and exacerbate the stress of raising a child alone, increasing the risk of a depressive state. It is therefore important to explore the relationship between mother and child’s father during the perinatal period and, if strained, prompt the screening of the mother for PND.

In this study, almost one-third of participants screened positive for a risk of suicide or infanticide, consistent with that of a South African study conducted in KwaZulu-Natal.^27^ However, this proportion is far higher than reported in developed countries (32.5 vs. 20%).^25,28^ and reveals a worrisome potential for the risk of suicide /infanticide amongst South African women in the postnatal period. Addressing this is an immediate policy and clinical imperative in South African community obstetric and PHC. Screening for suicidal ideation needs to be done in all mothers at risk of PND, and more importantly, all mothers attending the postnatal clinic should be asked at least Question 10 of the EPDS – ‘the thought of harming myself has occurred to you’. This is more so, that scoring at least 1 in Question 10 of the EPDS confers up to 22 times a higher risk of being screened positive for PND. Although Question 10 of the EPDS is not specific for assessing suicidal ideation, it has shown good reliability and sensitivity.

### Strengths and limitations

Being cross-sectional in design, the study findings represent the situation at a point in time and do not infer causality between other variables and PND. In addition, the study utilised a screening and not diagnostic tool, and the findings in no manner reflect the prevalence of PND.

There is a possibility of selection bias because of the consecutive sampling methods. Also, mothers younger than 18 years of age were excluded, and this could have underrepresented the proportion screened positive for PND, given that teenage pregnancy accounts for greater than 10% of all pregnancies in most settings in South Africa.^14^

The questionnaire was researcher administered, and participants could have responded to some questions in a socially desirable manner, resulting in errors in outcome estimates.

Notwithstanding these potential limitations, this study found a high risk for depressive disorders in the postpartum period in a typical South African primary care setting with serious clinical and health policy implications.

## Conclusion

Considering that a high proportion of participants in this study screened positive for PND, there is a potentially high burden of depressive disorders in the peri-partum period in this study setting. There is therefore a need for routine screening for PND amongst women in the peri-partum period in primary care, particularly those with low educational attainment, history of contraception failure prior to the index pregnancy and poor relationship with the father of the index child.
