# Erratum: Prevalence and risk factors associated with postnatal depression in a South African primary care facility

**DOI:** 10.4102/phcfm.v13i1.3179

**Published:** 2021-12-09

**Authors:** Nyundu S.J. Phukuta, Olufemi B. Omole

**Affiliations:** 1 Division of Family Medicine, Department of Family Medicine, Faculty of Health Sciences, University of The Witwatersrand, Johannesburg, South Africa

In the version of this article initially published, Phukuta NSJ, Omole OB. Prevalence and risk factors associated with postnatal depression in a South African primary care facility. Afr J Prm Health Care Fam Med. 2020;12(1), a2538. https://doi.org/10.4102/phcfm.v12i1.2538, the authors’ affiliation was given incorrectly in the ‘Affiliation’ section. The correct affiliation should be ‘Division of Family Medicine, Department of Family Medicine, Faculty of Health Sciences, University of The Witwatersrand, Johannesburg, South Africa’ instead of ‘Division of Family Medicine, Department of Family Medicine, Faculty of Sciences, University of The Witwatersrand, Johannesburg, South Africa’.

This correction does not alter the study’s findings of significance or overall interpretation of the study’s results. The publisher apologises for any inconvenience caused.

